# Incidence and Risk of QTc Interval Prolongation among Cancer Patients Treated with Vandetanib: A Systematic Review and Meta-analysis

**DOI:** 10.1371/journal.pone.0030353

**Published:** 2012-02-17

**Authors:** Jiajie Zang, Shunquan Wu, Lei Tang, Xudong Xu, Jie Bai, Caicui Ding, Yue Chang, Long Yue, Enming Kang, Jia He

**Affiliations:** 1 Department of Health Statistics and Center of Evidence-based Medicine, Second Military Medical University, Shanghai, China; 2 Cardiology Department, Changhai Hospital, Second Military Medical University, Shanghai, China; 3 Department of Geriatrics, Changhai Hospital, Second Military Medical University, Shanghai, China; 4 Library of First Hospital, Peking University, Beijing, China; 5 Department of Endocrinology, Changhai Hospital, Second Military Medical University, Shanghai, China; University of Modena and Reggio Emilia, Italy

## Abstract

**Background:**

Vandetanib is a multikinase inhibitor that is under assessment for the treatment of various cancers. QTc interval prolongation is one of the major adverse effects of this drug, but the reported incidence varies substantially among clinical trials. We performed a systematic review and meta-analysis to obtain a better understanding in the risk of QTc interval prolongation among cancer patients administered vandetanib.

**Methodology and Principal Findings:**

Eligible studies were phase II and III prospective clinical trials that involved cancer patients who were prescribed vandetanib 300 mg/d and that included data on QTc interval prolongation. The overall incidence and risk of QTc interval prolongation were calculated using random-effects or fixed-effects models, depending on the heterogeneity of the included studies. Nine trials with 2,188 patients were included for the meta-analysis. The overall incidence of all-grade and high-grade QTc interval prolongation was 16.4% (95% CI, 8.1–30.4%) and 3.7% (8.1–30.4%), respectively, among non-thyroid cancer patients, and 18.0% (10.7–28.6%) and 12.0% (4.5–28.0%), respectively, among thyroid cancer patients. Patients with thyroid cancer who had longer treatment duration also had a higher incidence of high-grade events, with a relative risk of 3.24 (1.57–6.71), than patients who had non-thyroid cancer. Vandetanib was associated with a significantly increased risk of all-grade QTc interval prolongation with overall Peto odds ratios of 7.26 (4.36–12.09) and 5.70 (3.09–10.53) among patients with non-thyroid cancer and thyroid cancer, respectively, compared to the controls.

**Conclusions/Significance:**

Treatment with vandetanib is associated with a significant increase in the overall incidence and risk of QTc interval prolongation. Different cancer types and treatment durations may affect the risk of developing high-grade QTc interval prolongation.

## Introduction

Vandetanib is a multikinase inhibitor that is currently under assessment for the treatment of a number of solid tumours. It targets key signalling pathways in cancer by inhibiting vascular endothelial growth factor receptor (VEGFR)-dependent tumour angiogenesis, and epidermal growth factor receptor (EGFR), and rearranged during transfection (RET)-dependent tumour cell proliferation and survival [1], [2].

Clinical benefits from the administration of vandetanib single agent were observed in a phase II clinical trial, and durable objective partial responses and disease control were observed among patients with advanced or metastatic hereditary medullary thyroid cancer (MTC) [3]. A phase III randomised controlled trial (ZETA) also demonstrated a 54% reduction in the risk of disease progression among MTC patients treated with vandetanib compared to placebo [4]. In April 2011, the U.S. Food and Drug Administration (FDA) approved vandetanib as an orphan drug that could be used to treat MTC unsuitable for surgical resection or metastatic MTC [5].

The efficacy of vandetanib was also observed in the treatment of patients with advanced non-small-cell lung cancer (NSCLC) when concomitantly administered with docetaxel [6]. Compared with gefitinib, vandetanib also demonstrated significant prolongation of progression free survival (PFS) [7]. An open-label phase II study (ZACTHYF) that assessed the benefit of vandetanib for patients with locally advanced or metastatic papillary or follicular thyroid cancer also showed significantly improved PFS compared with placebo (11.0 months *vs.* 5.8 months) [8]. The number of trials evaluating the benefit of vandetanib for the treatment of colorectal cancer [9], prostate cancer [10], hepatocellular carcinoma [11], and many other cancers has recently increased.

However, as with many other therapeutic drugs, vandetanib is associated with substantial side effects. Diarrhoea, nausea, rash, and hypertension are the most commonly reported adverse events when patients are prescribed vandetanib treatment. QTc interval prolongation is a major adverse effect that has been noted in trials; it is associated with a high risk of ventricular arrhythmias (e.g., torsade de pointes [TdP], syncope, and sudden death) [12], [13]. Thus, it is essential that doctors as well as patients receiving vandetanib therapy recognize and manage the risks for QTc interval prolongation.

Nevertheless, the incidence of QTc interval prolongation varies across clinical trials, and ranges from 5.1% to 44.4% [14], [15]; the overall risk of QTc interval prolongation in the patients compared with that in controls is unclear because of the limited sample sizes in each trial. Therefore, we sought to fully investigate the incidence and relative risk of QTc interval prolongation among patients administered vandetanib.

## Methods

### Search strategy

We searched the Pubmed (data from 1966 to April 2011), Embase (data from 1980 to April 2011) and the Cochrane Library electronic databases. Keywords included in the search were ‘vandetanib’, ‘ZD6474’, ‘cancer’, and ‘QTc’. The search was restricted to clinical trials and articles published in English. Proceedings for the annual meetings of the American Society of Clinical Oncology (ASCO) and the European Society of Medical Oncology (ESMO) (from 2001 to April 2011) were searched manually using the same keywords. Additionally, we searched the clinical trial registration website (http://www.ClinicalTrials.gov) to obtain information on the registered randomised controlled trials (RCTs). We also reviewed the reference lists of the original and review articles to identify relevant studies.

### Study selection and data collection

Two reviewers (JJZ and SQW) independently assessed the eligibility of the articles and abstracts identified by the search, and discrepancies were resolved by consensus. Since the daily dose of vandetanib approved by the FDA is 300 mg/d [5], we assessed the risk of QTc interval prolongation with vandetanib at this dose to ensure clinical significance. Because of the dosage variations and limited sample sizes in phase I trials, we excluded these trials from the analysis. Only Phase II and III clinical trials in which only vandetanib was administered at the defined dose were included. We analysed studies that fulfilled the following criteria: prospective clinical trials in patients with cancer; participants assigned to treatment with only vandetanib at a dosage of 300 mg/d; and availability of safety data related to QTc interval prolongation.

Data extraction was completed independently by 3 reviewers (YC, EMK, and LY) who used standardized data-collection forms. For each study that fulfilled the criteria, we extracted the following information: first author's name; year of publication; treatment arms; number of enrolled patients; number of patients in the treatment and control groups (when available); and adverse outcomes of interest (QTc interval prolongation).

### Clinical endpoints

QTc interval prolongation was recorded according to version 2 Common Terminology Criteria (CTC) or version 3 of the Common Terminology Criteria for Adverse Events (CTCAE) [16]. The upper limit value for QT/QTc interval prolongation was 0.48 s in version 2 CTC and 0.45 s in version 3 of CTCAE. Trials that used version 2 criteria to identify QTc interval prolongation might miss some events since patients whose QTc interval ranged from 0.45 s to 0.48 s were not recorded. However, if we only included the trials that used the new criteria, information would have been missed. Therefore, we categorized the criteria as follows: grade 1, QTc interval >0.45–0.47 s or asymptomatic, not requiring treatment; grade 2, QTc interval >0.47–0.50 s, ≥0.06 s above baseline or symptomatic, but not requiring treatment; grade 3, QTc interval >0.50 s or symptomatic and requiring treatment; and grade 4, QTc interval >0.5 s with life-threatening signs or symptoms (e.g., arrhythmia, congestive heart failure, hypotension, shock, syncope, TdP). We included all incidences of QTc interval prolongation of grade 1 or above in our analysis.

### Statistical analysis

We used version 2 of the Comprehensive Meta Analysis programme for all statistical analyses. The data of the number of patients with all grades and high grades (grade 3 and grade 4) of QTc interval prolongation and of the number of patients receiving vandetanib were extracted from the adverse events outcomes. For each study, we derived the proportion and 95% confidence interval (CI) of patients with QTc interval prolongation. For studies with a control group in the same trial, we also calculated and compared the relative risk (RR) of QTc interval prolongation.

Because thyroid functional disorders can lead to arrhythmia, which may prolong the QTc interval [17], and because patients with thyroid cancer have a longer treatment duration, which may also increase the risk of developing QTc interval prolongation compared to patients with non-thyroid cancer, we pooled results to create independent non-thyroid cancer and thyroid cancer groups. The study quality was assessed using the 5-point scale Jadad score [18]. A trial with a score of 3 or above was regarded as high quality.

For the meta-analysis, we used both fixed-effects and random-effects models. In each meta-analysis, the χ^2^ and I^2^ values were first calculated to assess the heterogeneity of the included trials [19]; p<0.10 for the χ^2^ test and I^2^<25% were interpreted as signifying low-level heterogeneity. When there was no statistically significant heterogeneity, a pooled effect was calculated using a fixed-effects model; otherwise, a random-effects model was employed. To calculate the pooled incidence, an inverse variance statistical method was used. To calculate the pooled RR, if the event rate was above 1%, we used the Mantel-Haenszel statistical method; otherwise, Peto odds ratios (ORs) were used to combine the RRs when events were rare. In this context, estimates of odds and risks are nearly identical, and both results can be interpreted as ratios of probabilities [20]. Funnel plots [21] and Egger's test [22] were also employed to assess the probability of publication bias. A two-tailed p<0.05 was deemed statistically significant.

### Role of the funding source

The sponsors of the study had no role in the study design, data collection, data analysis, data interpretation or in the writing of the report. The corresponding author had full access to all of the data in the study and had final responsibility for the decision to submit the study for publication.

## Results

### Flow of included studies

**Figure 1:**
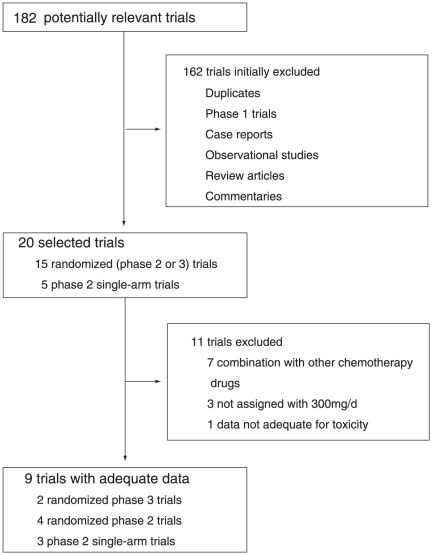
Selection process for trials.

A total of 182 potentially relevant trials with vandetanib were identified by the search strategy, 173 of which were excluded for the reasons shown in Figure 1. Nine trials met the inclusion criteria; 7 were published articles, and the rest were meeting abstracts. A total of 2,188 patients were available for the meta-analysis. Six trials were RCTs with a control arm, and 3 were single-arm trials.

### Study characteristics

Table 1 shows the characteristics of the individual trials. The types of cancer included small-cell lung cancer (SCLC) [23], advanced NSCLC [7], [14], [15], [24], thyroid cancer [4], [8], and breast cancer [25]. The incidence analysis included all the patients in the 9 trials. Median treatment duration ranged from 1.8 to 24.0 months and median progression-free survival ranged from 1.6 to 27.9 months. For calculation of the RRs, 6 RCTs were pooled; 1,134 patients were assigned to the drug group (vandetanib, 300 mg/d) and 976 were assigned to the control or placebo arms. Jadad scale was used to assess the quality of included trials. Overall, five trials had a Jadad score of 5, one scored 4, one scored 3 and two scored 2. Formal critical appraisal of the 9 trials indicated that the quality was high in 7 trials (Jadad score≥3) and low in two trial (Table 1). One of the trials with a Jadad score of two was single-armed trial, and another one was a phase 2 cohort study.

**Table 1:** Characteristics of clinical trials and patients included in the meta-analysis.

Trials	Phase	Histology	Treatment arms	Patients number	Median age	Median treatment(months)	Median PFS(months)	Jadad Score
Aronold(2007)	2	SCLC	vandetanib 300 mg/d	53	56.9	1.8	2.7	5
			Placebo	54	62.4	3.0	2.8	
Heymach(2008)	2	NSCLC	vandetanib 300 mg/d	73	63.0	NR	2.9	3
			Placebo+PC	52	59.0	NR	5.8	
Natale(2009)	2	NSCLC	vandetanib 300 mg/d	83	63.0	NR	2.8	5
			gefitinib 250 mg/d	85	61.0	NR	2.0	
Natale(2011)	3	NSCLC	vandetanib 300 mg/d	623	61.0	2.3	NR	5
			Erlotinib 150 mg/d	617	61.0	2.2	NR	
Miller(2005)	2	Breast cancer	vandetanib 300 mg/d	24	50.5	NR	1.6	2
Kiura(2008)	2	NSCLC	vandetanib 300 mg/d	18	61.0	NR	3.1	5
Wells(2010)	2	Advanced MTC	vandetanib 300 mg/d	30	49.0	18.8	27.9	2
Leboulleux(2010)	2	Advanced DTC	vandetanib 300 mg/d	72	63.0	18.9	11.0	4
			Placebo	73	63.0	19.5	5.8	
Wells(2011)	3	Advanced MTC	vandetanib 300 mg/d	231	53.0	24.0	>22.6	5
			Placebo	100	53.0	24.0	16.4	

NSCLC:non-small-cell lung cancer; SCLC:small-cell lung cancer; PC:paclitaxel and carboplatin; MTC:medullary thyroid cancer; DTC:differentiated thyroid cancer.

### Quantitative data synthesis

**Figure 2:**
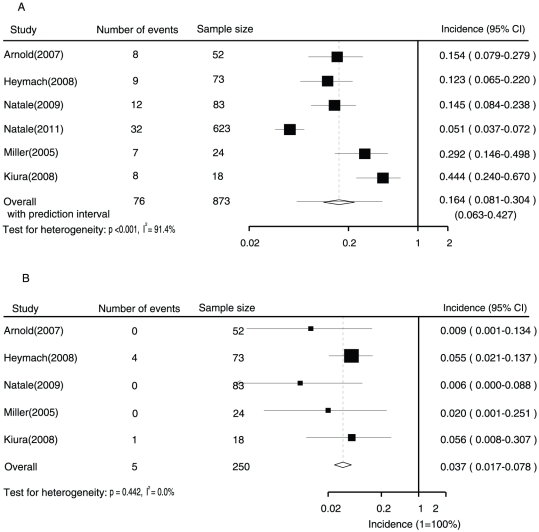
Forest plot for meta-analysis of incidence of all grade (A) and high-grade (B) prolonged QTc interval in patients with non-thyroid cancer who were assigned vandetanib.

Data relating to the incidence of all-grade QTc interval prolongation among 873 patients in the non-thyroid cancer group who were enrolled in 6 trials were available for analysis. The incidence ranged from 5.1 to 44.4%; the lowest incidence was noted in a phase III erlotinib-controlled randomised trial among patients with NSCLC, and the highest incidence was observed in a phase II single-arm trial among patients with breast cancer. The meta-analysis revealed the heterogeneity of the included studies (I^2^ = 91.4%, p<0.001). We explored the potential source of heterogeneity by analysing randomised controlled and non-randomised trials separately. The results showed that heterogeneity existed in the RCTs [7], [14], [23], [24], but not in the non-randomised trials [15], [25]. However, there was no compelling reason to exclude these trials. Analysis using a random-effects model revealed an overall incidence of 16.4% (95% CI, 8.1–30.4%) among non-thyroid cancer patients prescribed vandetanib. The prediction interval was calculated to be 0.063–0.427 (Figure 2A).

Data relating to high-grade QTc interval prolongation among patients in the non-thyroid cancer group were available for 250 patients enrolled in 5 trials. The incidence ranged from 0.6% to 5.6%, with the lowest incidence in a phase II gefitinib-controlled randomised trial among patients with NSCLC, and the highest in a phase II single-arm trial among patients with breast cancer. The overall incidence of high-grade QTc interval prolongation among non-thyroid cancer patients was 3.7% (95% CI, 1.7–7.8%; p for heterogeneity = 0.442, I^2^ = 0.0%), as determined using a fixed-effects model (Figure 2B).

**Figure 3:**
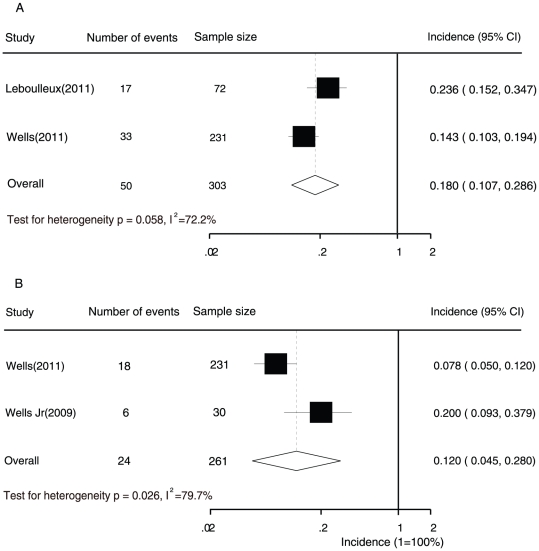
Forest plot for meta-analysis of incidence of all grade (A) and high-grade (B) prolonged QTc interval in patients with thyroid cancer who were assigned vandetanib.

We further analysed the incidence of QTc interval prolongation among patients with thyroid cancer. The overall incidence of all-grade and high-grade QTc interval prolongation was 18.0% (95% CI, 10.7–28.6%; p for heterogeneity = 0.058, I^2^ = 72.2%) and 12.0% (4.5–28.0; p for heterogeneity = 0.026, I^2^ = 79.7%), respectively (Figure 3), as determined by a random-effects model.

A difference was detected in the incidence of vandetanib-associated high-grade QTc interval prolongation (RR, 3.24; 95% CI, 1.57–6.71) between patients with thyroid cancer and those with a non-thyroid malignancy. There was no difference in the incidence of all-grade QTc interval prolongation (RR, 1.10, 0.67–1.87) between patients with the two types of cancer.

**Figure 4:**
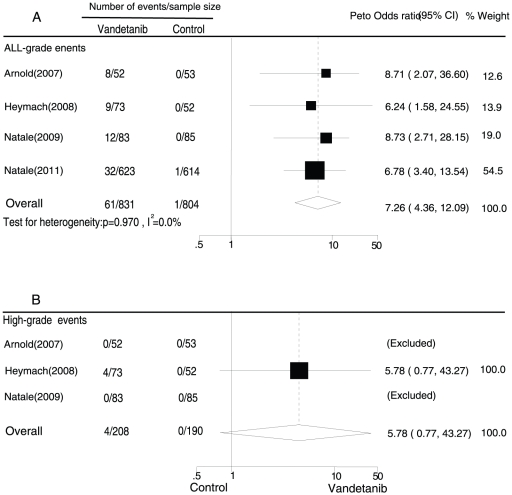
Peto Odds ratio of vandetanib-associated all grade (A) and high-grade (B) prolonged QTc interval versus control from the randomized controlled trials of patients with non-thyroid cancer.

The meta-analysis of the RR for QTc interval prolongation with vandetanib compared with controls was performed for the RCTs consisting of patients with both non-thyroid and thyroid cancer. Four and 3 RCTs were included in the analysis of all-grade and high-grade QTc interval prolongation, respectively, among non-thyroid cancer patients. Of the 4 RCTs included in the analysis of all-grade events, 1 trial used placebo as the control [23], another used placebo plus paclitaxel and carboplatin [24], another trial used gefitinib [7], and the final trial used erlotinib [14]. In all the trials, the incidence of QTc interval prolongation was low in the control groups (0/53, 0/52, 0/85, and 1/614, respectively). Since the event rate in the control group of each trial was lower than 1%, we used Peto one-step ORs. The overall Peto OR was 7.26 for vandetanib versus control for all-grade QTc interval prolongation among non-thyroid cancer patients (95% CI, 4.36–12.09; p for heterogeneity = 0.970, I^2^ = 0.0%), as calculated using a fixed-effects model (Figure 4A). Three RCTs were analysed for high-grade events [7], [23], [24]. However, the event rates in both the treatment and control groups were zero in 2 trials [7], [24]. Thus, only 1 trial [23] was included in the meta-analysis. The overall Peto OR was 5.78 for vandetanib versus control for high-grade QTc interval prolongation among non-thyroid cancer patients (0.77–43.27) (Figure 4B). Thus, vandetanib was associated with a significantly increased risk of all-grade QTc interval prolongation among patients with non-thyroid cancer compared with those who were not assigned vandetanib.

**Figure 5:**
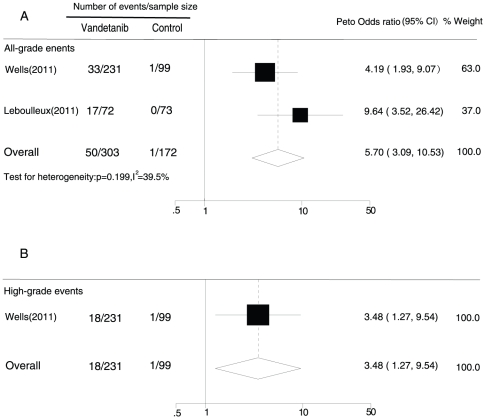
Peto Odds ratio of vandetanib-associated all grade (A) and high-grade (B) prolonged QTc interval versus control from the randomized controlled trials of patients with thyroid cancer.

Two RCTs [4], [8] were included in the analysis of all-grade QTc interval prolongation among thyroid cancer patients, and one [8] was excluded from the analysis of high-grade events. Both trials used placebo as a control. The overall Peto OR was 5.70 (95% CI, 3.09–10.53; p for heterogeneity = 0.199, I^2^ = 39.5%) and 3.48 (1.27–9.54) for vandetanib versus control for all-grade and high-grade QTc interval prolongation, respectively, among thyroid cancer patients, as calculated using a fixed-effects model (Figure 5).

No evidence of publication bias was detected for the incidence or Peto ORs of QTc interval prolongation events (all-grade and high-grade) by either funnel plots or Egger's tests (data not shown).

TdP occurred in 1 patient in a phase III erlotinib-controlled randomised trial; the patient recovered without sequelae after vandetanib was discontinued.

## Discussion

To the best of our knowledge, this is the first meta-analysis to investigate the overall risk of QTc interval prolongation associated with vandetanib in cancer patients and to find the differences in the risk between patients with thyroid cancer and those with a non-thyroid malignancy. The meta-analysis was based on 9 trials and 2,188 patients.

We noted that the overall incidence of all-grade and high-grade QTc interval prolongation with vandetanib (300 mg/day) was 16.4% (95% CI, 8.1%–30.4%) and 3.7% (1.7%–7.8%), respectively, among patients with non-thyroid cancer, and 18.0% (10.7–28.6%) and 12.0% (4.5–28.0%), respectively, among patients with thyroid cancer. The prediction interval for the incidence of all-grade QTc interval prolongation among patients with non-thyroid cancer who were assigned vandetanib was calculated as 0.063–0.427. This interval shows that at least 95% of the individual study settings have an incidence of all-grade QTc interval prolongation ranging from 6.3% to 42.7%.

The prevalence of borderline QTc interval prolongation ranged from 14% to 15% during treatment of oncology patients observed in a previous study, whereas arsenic trioxide, which can prolong the QTc interval, was associated with an incidence of 68.8% [26]–[29]. Some non-antiarrhythmic drugs such as cisapride [13] (a drug used to treat gastroesophageal reflux) and terfenadine [30] are often associated with a much higher incidence of QTc interval prolongation. Compared with the incidence of other drugs that cause a prolonged QTc interval, the incidence of all-grade QTc interval prolongation associated with vandetanib is moderate.

The risk of drug-induced catastrophic ventricular arrhythmia is of great interest to the FDA for the development of oncology interventions. The most concerning issue is the incidence of TdP, a polymorphic arrhythmia that can lead to sudden death. QTc interval prolongation is one of the most important risk factors to induce this life-threatening consequence. Much of the data on treatment-induced TdP are derived from patients with congenital long QT syndrome (LQTS), where the risk of TdP appears to be greater if the QTc interval is >500 ms [31] (this would be defined as high-grade QTc interval prolongation in our study). Intensive monitoring and management of high-grade QTc interval prolongation are crucial for patient safety. One patient observed in a trial [14] that primarily concerned treatment of NSCLC developed a TdP; however, the patient recovered after discontinuation of vandetanib. In future, the risks associated with vandetanib should be weighed against its efficacy when vandetanib is used in clinical practice.

Further exploratory analyses found a significant difference in the incidence of vandetanib-associated high-grade QTc interval prolongation (RR 3.24, 95% CI 1.57–6.71) between patients with thyroid cancer and those with a non-thyroid malignancy. Thus, clinicians and patients need to know that there are different risks for different diseases.

On one hand, the variability in the incidence of high-grade QTc interval prolongation in the different cancer types may be due to variations in treatment duration. The median treatment period for a non-thyroid malignancy ranged from 1.8 to 3.0 months. However, the median duration of thyroid cancer therapy was >18.8 months.

On the other hand, QTc interval prolongation has also been detected in patients with hypothyroidism and subclinical hypothyroidism (SH) [17], [32], [33], as well as in patients with high free thyroxine levels and hyperthyroidism [34]–[36]. Some individuals with thyroid cancer are more often associated with an abnormal thyroid function [17], [37], and are more prone to acquire prolonged QTc intervals. When administering a drug that potentially prolongs the QTc interval, thyroid cancer patients might be more vulnerable to the severe side effects of the longer QTc interval than those with a non-thyroid malignancy.

The analysis of data from the RCTs also revealed significant 5.70- and 7.26-fold increases in the Peto ORs of all-grade QTc interval prolongation among patients with thyroid cancer and those with non-thyroid malignancies, respectively, compared with controls. The Peto ORs of high-grade QTc interval prolongation were also assessed separately. There was a 3.48-fold increase in the Peto OR for high-grade QTc interval prolongation among patients with thyroid cancer. No difference was detected among non-thyroid cancer patients. Nevertheless, the number of trials eligible for evaluation of the risk of high-grade QTc interval prolongation in these 2 cancer types was small and more trials should be included to evaluate the true risk of high-grade QTc interval prolongation in the future.

One of the strengths of the present meta-analysis is that we quantitatively identified the incidence of QTc interval prolongation by using data from trials of patients who underwent vandetanib therapy for different cancers. Vandetanib was approved by the FDA for the treatment of patients with advanced MTC [5]. It has also been reported to be effective in the treatment of other cancers [6], [8], [38]. Thus, it is worthwhile to devote resources toward a detailed evaluation of its adverse effects because it might be widely used in clinical practice. Besides, a detailed analysis of the adverse effects would be warranted if the information on potential harm appears to be essential for guiding the decisions of clinicians, consumers, and policymakers. Many of the RCTs in our study had extremely few patients that the data were not reliable for detecting meaningful differences in the incidence of adverse events. However, this meta-analysis combined data from a number of trials and thus had greater statistical reliability. Moreover, consistent results were found with respect to the sensitivity analyses, and no evidence of publication bias was found.

Our study considered the difference in the incidence of QTc interval prolongation in association with both different cancer types and treatment durations. It is nearly impossible to perform a trial that primarily compares the different risks of one adverse event between different cancers. However, it is useful for drug agencies and doctors to determine the administration of this drug in different diseases or for different treatment periods.

A limitation of this study is that the findings are not based on individual patient data as those of many other meta-analyses are. The trials included in our study might have underestimated the incidence and RR of all-grade vandetanib-associated QTc interval prolongation because of the use of different versions of adverse event reporting criteria; the true incidence and RR might therefore be higher. Some trials adopted CTCAE version 2 to record adverse events, where a prolonged QTc interval was identified when the QTc interval was >0.48 s. Other trials employed CTCAE version 3, in which a prolonged QTc interval was identified when the QTc interval was >0.45 s. The former criterion is less strict when reporting QTc interval prolongation and patients with QTc intervals ranging from 0.45 s to 0.48 s would thus not have been recorded as having an adverse event [16]. However, if we eliminate the trials that used CTCAE version 2 as a criterion, much of the information would be missed, although we might obtain a higher incidence and RR. As for the criteria for high-grade QTc interval prolongation in the 2 versions, they are similar due to their clinical severity (that is, they require treatment or have life-threatening consequences).

We only assessed the risk of QTc interval prolongation with vandetanib (300 mg/d) in this study. The risk at other doses or in combination with other anticancer drugs was not evaluated. Physicians should carefully interpret these results when they apply them in clinical practice.

In conclusion, this study has shown that vandetanib is associated with a significant increased risk of developing QTc interval prolongation. The incidence of high-grade QTc interval prolongation differs between thyroid cancer patients who require a longer treatment duration and patients with non-thyroid malignancies who require a shorter treatment period. The prolonged QTc interval and other cardiac side effects of vandetanib require thorough post-market surveillance and reporting.
